# ABO blood groups and the risk of SARS-CoV-2 infection

**DOI:** 10.1007/s00709-022-01754-1

**Published:** 2022-04-01

**Authors:** Jörn Bullerdiek, Emil Reisinger, Birgit Rommel, Andreas Dotzauer

**Affiliations:** 1 Institute for Medical Genetics, University of Rostock, University Medicine, Ernst-Heydemann-Strasse 8, 18057 Rostock, Germany; 2 Human Genetics, University of Bremen, Leobener Strasse 2, 28359 Bremen, Germany; 3 Department of Tropical Medicine and Infectious Diseases, Ernst-Heydemann-Strasse 6, 18055 Rostock, Germany; 4 Laboratory of Virus Research, University of Bremen, Leobener Strasse 6, 28359 Bremen, Germany

**Keywords:** ABO blood groups, SARS-CoV-2 infection, COVID-19, Severity

## Abstract

There is no doubt that genetic factors of the host play a role in susceptibility to infectious diseases. An association between ABO blood groups and SARS-CoV-2 infection as well as the severity of COVID-19 has been suggested relatively early during the pandemic and gained enormously high public interest. It was postulated that blood group A predisposes to a higher risk of infection as well as to a much higher risk of severe respiratory disease and that people with blood group O are less frequently and less severely affected by the disease. However, as to the severity of COVID-19, a thorough summary of the existing literature does not support these assumptions in general. Accordingly, at this time, there is no reason to suppose that knowledge of a patient’s ABO phenotype should directly influence therapeutical decisions in any way. On the other hand, there are many data available supporting an association between the ABO blood groups and the risk of contracting SARS-CoV-2. To explain this association, several interactions between the virus and the host cell membrane have been proposed which will be discussed here.

**Supplementary Information:**

The online version contains supplementary material available at 10.1007/s00709-022-01754-1.

## Genetics and biochemistry of the ABO blood groups

Among the mammalian species, the polymorphic ABO blood group system based on carbohydrate antigens is restricted to primates (for review, see Ségurel et al. 2012). The first description of the genetics of this system dates back to 1910 when von Dungern and Hirschfeld were able to apply the basic principles of Mendelian inheritance to familial segregation of ABO blood groups and to predict the forensic relevance of their findings (Supplemental Fig. 1). Phenotypically, the A and B behave co-dominantly over the recessive O. This system is the first genetic polymorphism described in humans, making von Dungern’s and Hirschfelds’s paper one of the “founder documents” of human genetics at all. Soon after this initial report, the findings were the basis for the first study of genetic variation in human populations carried out by the Hirschfelds during the First World War and published in 1919 (Hirschfeld and Hirschfeld 1919, l.c. Bodmer, 2015). The expression of ABO blood groups depends on the activity of a galactosyltransferase encoded by the ABO gene mapping to chromosomal band 9q34.2. In the absence of the active enzyme, the H antigen encoded by the *FUT1* gene is not converted into the A or B antigen. Depending on the activity of the co-dominant alleles encoding the active transferase enzyme, H antigen is converted, resulting in A, B, or AB blood group making H antigen the precursor of either A or B. Thus, four resulting phenotypes can be distinguished, i.e., A, B, AB, and O.

As to the biochemistry, the initial step on red blood cells is catalyzed by the gene product of *FUT1*: a precursor becomes transferred to the H-active structure, a terminal α-L-fucosyl-(1,2)-β-D-galactosyl moiety. The H-active structure then serves as the precursor of either the A or the B antigen by the activities of the transferases through addition of either N-acetylgalactosamine in α-(1 → 3) (A antigen) or transfer of D-galactose (B antigen). Accordingly, depending on the cell surface glycans, four resulting phenotypes can be distinguished, i.e., A, B, AB, and O. Of note, O can also result from the absence of H (commonly referred to as the “Bombay phenotype”) (Bhende et al. 1952). A highly similar substrate specificity as that of *FUT1* is displayed by the *FUT2* gene expressed in cells of secretory glands which produce a soluble form of the H antigen (secretor phenotype).

In addition to erythrocytes, ABO oligosaccharide antigens are expressed on a variety of different cell types such as respiratory epithelial cells as well as mucosal and gut epithelium, vascular endothelial cells (Alvarez-Fernandez and Carretero-Albiñana 1991; Bloom et al. 1993; for review, see Franchini and Liumbruno 2013), and even some benign and malignant tumors (Woltering et al. 1983; Itzkowitz 1992). Accordingly, these antigens are commonly referred to as histo-blood antigens.

## The ABO blood groups and infectious diseases

An association of blood groups with several infectious diseases has been described and may in part explain regional differences in infectious disease distribution. Well-known examples are associations of ABO blood types with infectivity or disease severity for several bacterial infections (for review, see Cooling 2015) as, e.g., the association between cholera severity and the O phenotype, the reason of which has been a matter of debate for many years (Barua and Paguio 1977; Harris and LaRocque 2016).

In contrast to other infectious diseases, viral infection always requires uptake of the viral agent by its target cells. Prior to its entry, the virus has to attach to the cell membrane. This latter step usually also involves molecules on the membrane of the host cell. For example, an interplay between glycans and glycan-binding proteins decorated on the surface of the virus and its host cell, respectively, is known to play a role during viral attachment (for review, see Raman et al. 2016). For obvious reasons, the genetics of the host can influence this interplay thereby contributing to inherited differences of viral susceptibility. A well-known example is the ABO blood group system in humans and primates which is often discussed as a risk modifier for a variety of viral diseases. Accordingly, an association of ABO types with viral infections is in some cases documented or has been suggested. This association might be connected with the broad spectrum of expression of the ABO antigens in various cell types. For example, several studies have revealed an association between Norovirus (NV) infection and ABO blood groups or the secretor phenotype, respectively (e.g., Hutson et al. 2002; Nordgren et al. 2013, for review, see Ruvoën-Clouet et al. 2013 and Liao et al. 2020). The earliest finding was obtained among participants of the NV challenge study, which implied that individuals with an O phenotype were more likely to get infected with NV. In contrast, persons with a B histo-blood group antigen had a decreased risk to become infected and to have symptomatic disease (Hutson et al. 2002). As an explanation, it has been suggested by the same group that carbohydrate antigens in the gut are a previously unrecognized factor in NV pathogenesis (Hutson et al. 2003). An example of a more severe course of a viral disease in correlation with the blood group type is the association of blood group AB with severe dengue disease, when associated with a secondary infection by certain dengue virus serotypes (Kalayanarooj et al. 2007). The reasons for this have not yet been clarified. Herein, only those associations that are related to the subject are outlined because it is not the aim of this paper to give a more or less complete review on associations between ABO blood groups and viral diseases. For a comprehensive review on blood groups and infectious diseases, readers are referred to Cooling (2015).

From the Hongkong outbreak of SARS-CoV-1, data obtained on a group of health care workers who were exposed to one index patient suggested an association between the ABO blood groups and infection. People with A, B, or AB were more likely to become infected compared to a relative resistance for blood group O (odds ratio (OR) of 0.18) (Chen et al. 2005). As an explanation, it has been hypothesized that the presence of ABO antibodies could block viral adhesion and potentially decrease the rate of infection, thereby offering a relative protection of people with O (Guillon et al. 2008). Accordingly, in vitro attempts were made to decorate the viral S (spike) protein by anti-A antibodies to block adhesion. Only plasma samples from O blood group individuals with high anti-A titers as determined by classical hemagglutination were found to be inhibitory in the cell adhesion assay (Guillon et al. 2008). A possible protection may thus depend on the antibody titer and additional effects of the secretor status can be suggested as well (Cooling 2015).

Akin to SARS-CoV-1, possible associations of the ABO blood groups with the severity of COVID-19 or different risks to become infected with SARS-CoV-2 have been discussed for a couple of months and have simultaneously gained a wide-spread interest in public media as well (Bullerdiek 2020c). Overall, the huge variety of these studies may make the possible COVID-19–ABO blood group association one of the most intensively studied associations between blood groups and an infectious disease. As to possible associations between either different probabilities of contracting SARS-CoV-2 or the severity of COVID-19, several explanations have been proposed addressing the presence of AB antibodies as delineated above for SARS-CoV-1 (see e.g., Yamamoto et al. 2021). Moreover, some authors recently suggested different degrees of protection (Li et al. 2020) or even therapeutical strategies based on ABO blood groups of COVID-19 patients (Sardu et al. 2020; AbdelMassih et al. 2020). This ongoing discussion makes an overview summarizing the currently available data reasonable. This review is addressing clinical/serological studies as well as genome-wide association studies comparing groups of differently affected subjects. In contrast, studies of other types mainly correlating the rate of SARS-CoV-2 infections or their severity with regional distribution of ABO blood groups have not been taken into consideration (e.g., Delanghe et al. 2021).

## ABO blood groups: association with SARS-CoV-2 infection or COVID-19 severity?

Though for obvious reasons all relevant papers available so far are less than 2 years old, a possible link between ABO blood groups already has been studied intensively in a variety of studies. The possible association of COVID-19 severity with ABO blood groups certainly ranks among the most investigated in the field of genetics of infectious diseases.

**Fig. 1:**
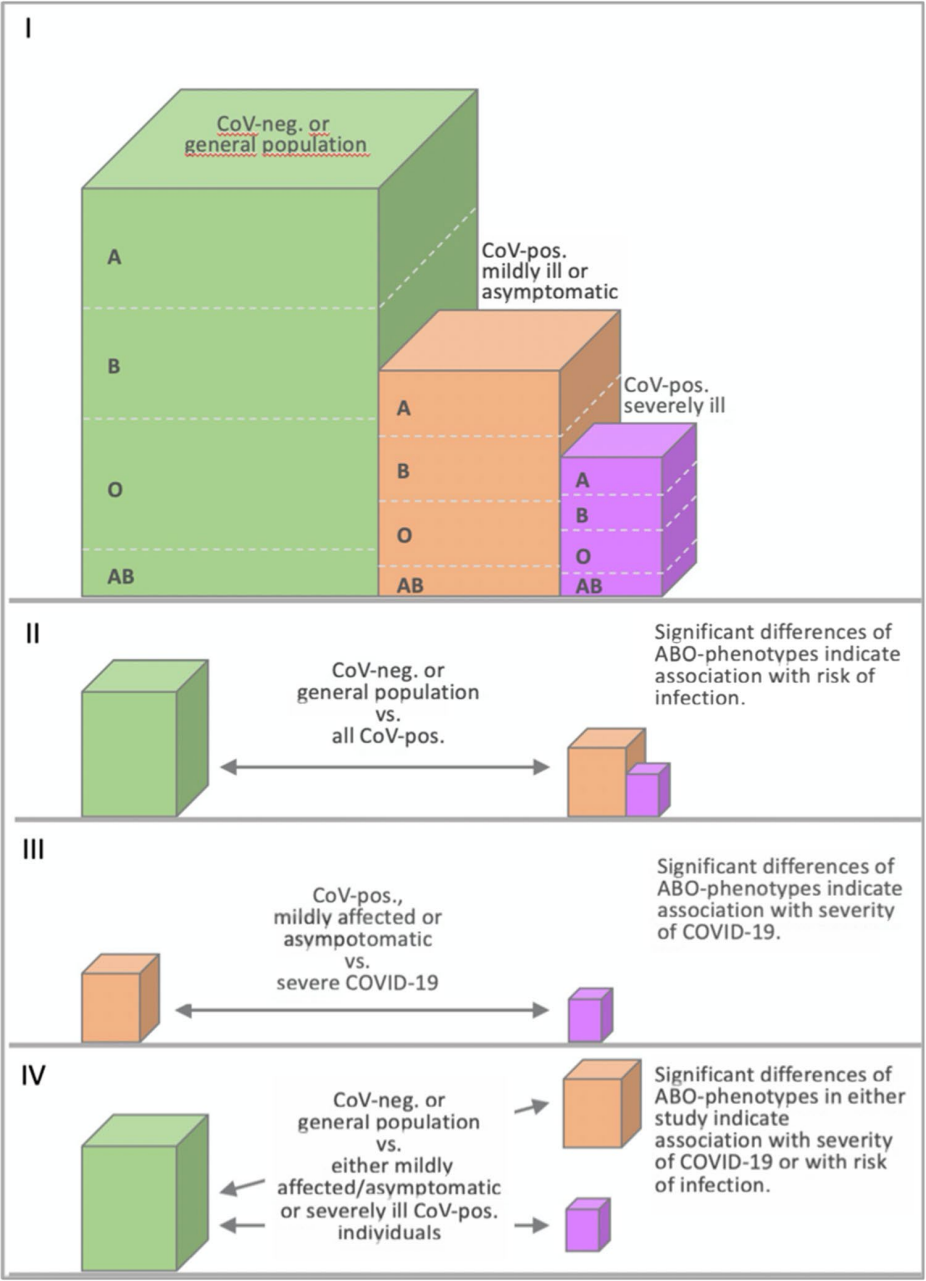
Scheme illustrating main types of association studies and their interpretation, as well as their limits. The size of the columns is only to illustrate the different groups and does not represent real percentage taken from the different studies. However, similar distinctions, e.g., underly the different evaluations as carried out by the COVID-19 host genetics initiative (COVID-19 host genetics initiative, 2021)

Early during the SARS-CoV-2 outbreak, an association between ABO blood groups and COVID-19 has been observed based on the results of a study on hospitalized Chinese COVID-19 patients from the Wuhan area (Zhao et al. 2020a, b, Table 1). In comparison to controls, the blood group distribution in the patient group differed significantly: The percentage of blood group A in COVID-19 patients was higher than that in the general population whereas that of blood group O in patients was lower than in the population. The same differences were also seen when comparing dead patients with the general population. The authors concluded that persons with blood group A had a significantly higher risk of infection than the average risk whereas the risk for people with O was decreased. The design of the study does not allow for a conclusion if the blood group may affect the risk of infection or the risk of a more severe course of the disease because no groups of patients/infected persons with a different degree of severity were compared (Fig. 1). Nevertheless, though recommending caution to use their study to guide clinical practice, Zhao et al. (2020a, b) have speculated on possible clinical consequences of their findings as introducing ABO typing into the routine part of management of COVID-19 and considering a more aggressive treatment. Of note, however, Dzik et al. (2020), when re-analyzing the data by Zhao et al., did not find an association between ABO type and death among individuals hospitalized with COVID-19.

**Table 1:** For the sake of transparency, in case of preprints preceding a peer-reviewed paper in some cases, both have been included in this table even if a complete overlap of data was noticed. In case of more than one version of a preprint, the different versions have been included as separate entries if and when the number of cases or controls or their description, respectively, had been changed. Though in some cases, associations of Rh-blood groups and either the risk of infection or the risk of severe disease have been mentioned; this is only additional information in some cases and far from being representative. Of note, two main questionnaire-based studies have not been included (Shelton et al., 2020; El-Shitany et al., 2021). *CoV* + tested positive for SARS-CoV-2 (if not mentioned otherwise by qRT-PCR), *CoV − *tested negative for SARS-CoV-2 (if not mentioned otherwise by qRT-PCR), *CRRT* continuous renal replacement therapy, *CS* clinical/serological study, *ECMO* extracorporeal membrane oxygenation, *GWAS* genome-wide association study, *ICU* intensive care unit

Reference	Type of study/country	Cases/patients and controls	Conclusions
Al-Youha et al., 2021	CS Kuwait	3305 patients. Control: 3,730,027 anonymized individuals representing almost Kuwait’s entire population; data obtained from a national database	No association between severe COVID-19 and ABO blood group in a unique, unselected population but increased risk of pneumonia in group A, lower prevalence of blood group O in individuals infected with SARSCoV-2, and a higher prevalence of B and AB. No association between SARS-CoV-2 infection rates with blood group A or RhD group
Badedi et al., 2021	CS Saudi Arabia	323 Saudi adults with COVID-19 of whom 108 died, 215 recovered	No significant association between blood group and mortality risk
Barnkob et al., 2020	CS Denmark	Of 473,654 individuals tested, 7422 were CoV + , and 466,232 were CoV − . Of the CoV + individuals, 1951 were hospitalized and 5471 non-hospitalized and 550 deceased and 6872 alive	ABO type not a risk factor for hospitalization or death; blood group O associated with decreased risk for SARS-CoV-2 infection
Bhandari et al., 2020	CS USA	A total of 825 cases (admitted with confirmed COVID-19 infection by RT-PCR) and 396 controls (seen at the same institution during the calendar year of 2019) were included	No significant relationship of ABO blood groups with susceptibility and mortality
COVID-19 host genetics initiative, release 5, January 18, 2021 (see also Niemi et al., 2021)	GWAS	Several analyses with a total of 49,562 COVID-19 patients including a comparison of hospitalized COVID vs. not hospitalized COVID patients, total cases 5,773, total controls 15,497	Significant association of *ABO* locus with infection but no significant association of *ABO* locus with disease severity when comparing hospitalized COVID-19 vs. not hospitalized patients (B1_ALL_leave_23andme)
Ellinghaus et al., 2020 medRxiv preprint (see also Severe Covid-19 GWAS Group)	GWAS Italy and Spain	1980 patients with severe COVID-19 (all receiving oxygen) vs. 2381 controls including 998 randomly selected blood donors with no evidence of COVID-19 who were genotyped for the purpose of the study as well as 1383 individuals from previous GWAS studies before the Corona pandemic using the same genotyping array)	Compared to the controls, patients with blood group A were overrepresented whereas those with blood group O turned out to be underrepresented. Design of the study not allowing for a conclusion if blood group A may be associated with either a higher risk for SARS-CoV-2 infection or a higher risk for a severe disease
Gamal et al., 2021	CS Italy	1601 SARS-CoV-positive persons with known ABO blood group compared to 27,715 first time blood donors	Blood group A significantly overrepresented among SARS-CoV-2 positive patients. No significant association between blood groups O, B, and AB and the susceptibility to acquire SARS-CoV-2 infection
Göker et al., 2020	CS Turkey	186 CoV + patients (with PCR confirmed diagnosis of COVID-19) as well as 1881 healthy control individuals (blood donors) were included	No significant effect of ABO blood groups on the clinical outcome; blood group A increased susceptibility to infection and blood group O decreased susceptibility as compared to type A
Gómez et al., 2021	CS Spain	566 hospitalized patients (mean age 64.57 years, range 24–95; 65% male), 236 admitted to ICU, control: 300 healthy individuals of the same age range	A-group was a significant risk factor for developing a severe form of COVID-19 with ICU admission. Compared with healthy population controls, patients with COVID-19 requiring hospitalization showed no significantly different ABO-genotype frequencies
Hoiland et al., 2020	CS Canada	95 critically ill COVID-19 patients with ABO blood group data available included in the analyses	Critically ill COVID-19 patients with blood group A or AB: increased risk for requiring mechanical ventilation, CRRT, and prolonged ICU admission compared to patients with blood group O or B
Hultström et al., 2020	CS Sweden	64 CoV + patients from a critical care cohort compared to the blood type distribution in the population as a whole	Blood type A or AB associated with increased risk of requiring critical care or dying; the design of the study does not allow for a conclusion if blood group A may be associated with either a higher risk for SARS-CoV-2 infection or a higher risk for a severe disease
Kotila et al., 2021	CS Nigeria	302 patients including 297 with known blood group, 179 symptomatic, control: blood donor population	Blood group O is protective against COVID-19 infection while blood groups B and AB are risk factors; patients with anti-B (blood groups O and A) in their serum were less likely to be infected by the virus; patients with anti-A (blood groups O and B) were more likely to become symptomatic from the infection. No susceptibility of group A to the infection was found
Latz et al., 2020	CS USA	Of 1289 CoV + patients, 484 (37.5%) were admitted to hospital, 123 (9.5%) were admitted to the ICU, 108 (8.4%) were intubated, 3 (0.2%) required ECMO, and 89 (6.9%) died	Blood type not associated with risk of progression to severe disease requiring intubation or causing death, nor with higher peak levels of inflammatory markers
Leaf et al., 2020	CS USA	2033 critically ill patients with COVID-19 included. The expected distribution of ABO phenotype in each of the above race/ethnicity categories was estimated using data from 3.1 million blood donors in the USA	Among white patients, the observed distribution of ABO phenotypes differed from its expected distribution; this difference was primarily driven by patients with blood type A and 0; among black and Hispanic patients, the observed and expected distributions of ABO phenotypes were similar; the mortality rate was similar across ABO phenotypes in all race/ethnicity categories
Levi et al., 2020	CS Brazil	6457 CoV + individuals by either RT-PCR or antibody test compared to unaffected patients	ABO blood group without significant impact on the risk for SARS-CoV-2 infection
Li et al., 2020	CS China	265 hospitalized patients, 3694 controls	People with blood group A had a significantly higher risk of SARS-CoV-2 infection, whereas blood group O had a significantly lower risk. In dead patients, no differences between blood types
Majeed et al., 2021	CS Iraq	5668 COVID-19 patients (all PCR-positive) along with the same number of control samples	No evidence for association of blood types, including RhD, with death due to COVID-19 when adjustments were made for age, gender, and risk factors (see Fig. 2B)
Mendy et al., 2020	CS USA	428 COVID-19 patients including 192 patients (44.9%) were hospitalized and 101 (23.6%) had a severe form of the disease	ABO blood group was neither associated with hospitalization nor with disease severity
Muñiz-Diaz et al., 2021	CS Spain	965 COVID-19 patients were severely affected and transfused during their hospitalization	Individuals with blood group A have a higher risk of death than those with group O (OR 1.39, 95% CI: 1.03–1.86) or than those with non-A blood groups
Nauffal et al., 2021	CS USA	409 individuals who had ABO blood group data available were examined for frequency, management, and outcomes of arterial and venous thromboembolic complications	Blood group A to be associated with higher odds of major cardiovascular events but no association between blood group and all-cause mortality
Niemi et al., 2021, see COVID-19 host genetics initiative, 2021	GWAS Meta-analysis with data across 19 countries		Significant association of ABO locus with infection but no significant association of ABO locus with disease severity when comparing hospitalized COVID-19 vs. not hospitalized patients (B1_ALL_leave_23andme)
Niles et al., 2021	CS USA	276,536 females with matched SARS-CoV‐2 and ABO‐Rh results, 34,178 being tested positive, for 88,975 race/ethnicity provided	Rh positivity, independent of ABO blood group and race/ethnicity, was a risk factor for SARS‐CoV‐2 positivity. Blood type O is slightly protective against SARS‐CoV‐2 positivity. Once race/ethnicity has been considered; association between ABO/Rh and SARS‐CoV‐2 positivity was confirmed but greatly attenuated after factoring in race/ethnicity
Pairo-Castineira et al., 2020	GWAS UK	2244 critically-ill COVID-19 patients from 208 UK ICUs, ancestry-matched controls from the UK Biobank population study and results were confirmed in GWAS comparisons with two other population control groups: the 100,000 genomes project and Generation Scotland	The ABO locus was not genome-wide significant. A signal close to genome-wide significance at this locus in the combined meta-analysis suggests that this variant may be associated with susceptibility to COVID-19, but not critical illness
Pairo-Castineira et al., 2021	See Pairo-Castineira et al., 2020	See Pairo-Castineira et al., 2020	See Pairo-Castineira et al., 2020
Ray et al., 2020	CS Canada	7071 CoV + individuals including 1328 with severe COVID-19 or death. For comparison, the total group of 225,556 CoV + or CoV − patients was included	O and Rh − blood groups with a slightly lower risk for SARS-CoV-2 infection; when restricted to 7071 persons who tested positive for SARS-CoV-2, no association between blood group and the risk for severe illness or death; in contrast, when analyzing all 225,556 CoV + or CoV − patients, type O blood versus others was protective against SARS-CoV-2 positivity with or without severe illness or death
Roberts et al., 2020	GWAS USA	COVID-19 survey responses between April and May 2020 with accompanying genetic data from the AncestryDNA customer database. Susceptibility GWAS with 2417 cases (COVID-19 positive) and 14,993 controls (COVID negative). Susceptibility GWAS with 250 cases (COVID-19 positive reporting hospitalization) and 1967 controls (COVID-19 positive reporting no hospitalization)	Associations with severe COVID-19 near ABO locus (*p* < 0.05), but not with susceptibility
Sardu et al., 2020	CS Italy	Compared O vs*.* non-O blood group in hypertensive patients with COVID-19 infection (72 vs. 92 patients admitted to one hospital)	Non-O COVID-19 hypertensive patients have significantly higher values of pro-thrombotic indexes, as well as higher rate of cardiac injury and deaths compared to O patients; ABO blood type influences worse prognosis in hypertensive patients with COVID-19 infection
Severe Covid-19 GWAS Group et al., 2020 (see Ellinghaus et al., 2020)	GWAS	1980 patients with severe COVID-19 (all receiving oxygen) vs. 2381 controls (including 998 randomly selected blood donors who were genotyped for the purpose of the study. A total of 40 of these participants had evidence of the development of anti–SARS-CoV-2 antibodies, all of whom had mild or no COVID-19 symptoms as well as 1383 individuals from previous GWAS studies before the Corona pandemic)	Compared to the controls, patients with blood group A were overrepresented in the group of patients whereas those with blood group O turned out to be underrepresented. Design of the study not allowing for a conclusion if blood group A may be associated with either a higher risk for SARS-CoV-2 infection or a higher risk for a severe disease
Solmaz and Araç, 2020	CS Turkey	1667 COVID-19 patients compared to general population	Blood group O could be associated with decreased risk for SARS-CoV-2 infection; blood group A with increased risk for infection; blood group does not affect the course of the disease and is not associated with mortality
Tonon et al., 2021	CS France	172 patients admitted to ICU	ABO blood group type may not be related to the severity of SARS-CoV-2 infection or related death
Yaylaci et al., 2020	CS Turkey	397 patients treated due to COVID-19 infection	Most frequent blood type was A + ; Rh + blood group in all cases admitted to ICU and with death outcome: The Rh + blood group was found in a significantly high number of patients who were admitted to ICU; no relationship between mortality and Rh blood group. None of the comparative analyses of O, A, B, and AB groups with other blood groups revealed a significant relationship with ICU admission and mortality
Zalba Marcos et al., 2020	CS Spain	226 patients with (PCR-positive) COVID-19 admitted to the hospital, including 17.9% who were admitted to the ICU and 16.3% who died. Population ABO blood groups from donors as controls	Both respiratory complications and deaths without significant differences between blood groups; blood group associated with thrombotic complications and admission to the ICU: blood group B developed more thrombosis and required more admission to the ICU, with group O being the least admitted to ICU
Zhang et al., 2021	CS UK	Information on ABO blood group was available for 1713 patients who tested positive for COVID-19. Of these, 227 of 682 with blood type O and 365 of 1031 with other blood types were hospitalized	Similar percentage of positive tests among ABO blood groups. Participants with blood group O less likely to be hospitalized with COVID-19 after a positive test (33.3% versus 38.0%)
Zhao et al., 2020a version 1 and Zhao et al., 2020b version 2	CS China	2173 patients with COVID-19, 206 dead, control: 27,080 (population of the respective areas)	Blood group A associated with a higher risk for acquiring COVID-19; blood group O associated with a lower risk for infection; blood group A was associated with a higher risk of death compared with non-A groups
Zietz and Tatonetti, 2020a version 1 as of April 11	CS USA	682 CoV + individuals including 179 who were intubated and 80 who had died. Compared to 877 CoV-negative individuals	Blood group A associated with increased odds for infection; blood group O associated with decreased odds for infection; few individuals with AB blood groups were included (21 COV + , 47 COV −); no association between blood group and intubation or death
Zietz and Tatonetti, 2020b version 2 as of July 21		Study evaluating associations between blood groups and outcomes using four pairs of populations: COV + vs. COV − , COV + vs. general population (excluding those tested for SARS-CoV-2), COV + /intubated vs. COV + /not intubated, and COV + /deceased vs. COV + /alive	Significant associations between SARS-CoV-2 test results and both Rh (*p* = 0.00041) and ABO/Rh (*p* = 0.048) blood groups, though not for ABO alone. The only significant ABO blood group association was between blood group A and intubation (*OR* 0.762, 95% *CI* [0.620–0.937], *p* = 0.0099)
Zietz et al., 2020a version 3 as of September 10		Analyzed 14,112 individuals tested for SARS-CoV-2 with known blood type in the New York Presbyterian (NYP) hospital system including 2394 COV + individuals; associations between ABO and Rh blood types and infection, intubation, and death were evaluated; data are highly enriched for severely ill patients	Slightly increased infection prevalence among non-O blood types; risk of intubation decreased among A and increased among AB and B types, compared to type O; risk of death increased for type AB and decreased for types A and B. Self-assessment of the study: “blood type appears to have a consistent effect, though the magnitudes of these effects on risk of intubation or death are modest, and estimates have large uncertainties relative to their magnitudes; the relatively large estimated errors in our analysis also suggest modest effect sizes and that greater sample sizes or meta-analyses are needed to estimate these effects more precisely.”
Zietz et al., 2020b	CS USA	See Zietz et al., 2020a	See Zietz et al., 2020a

**Fig. 2:**
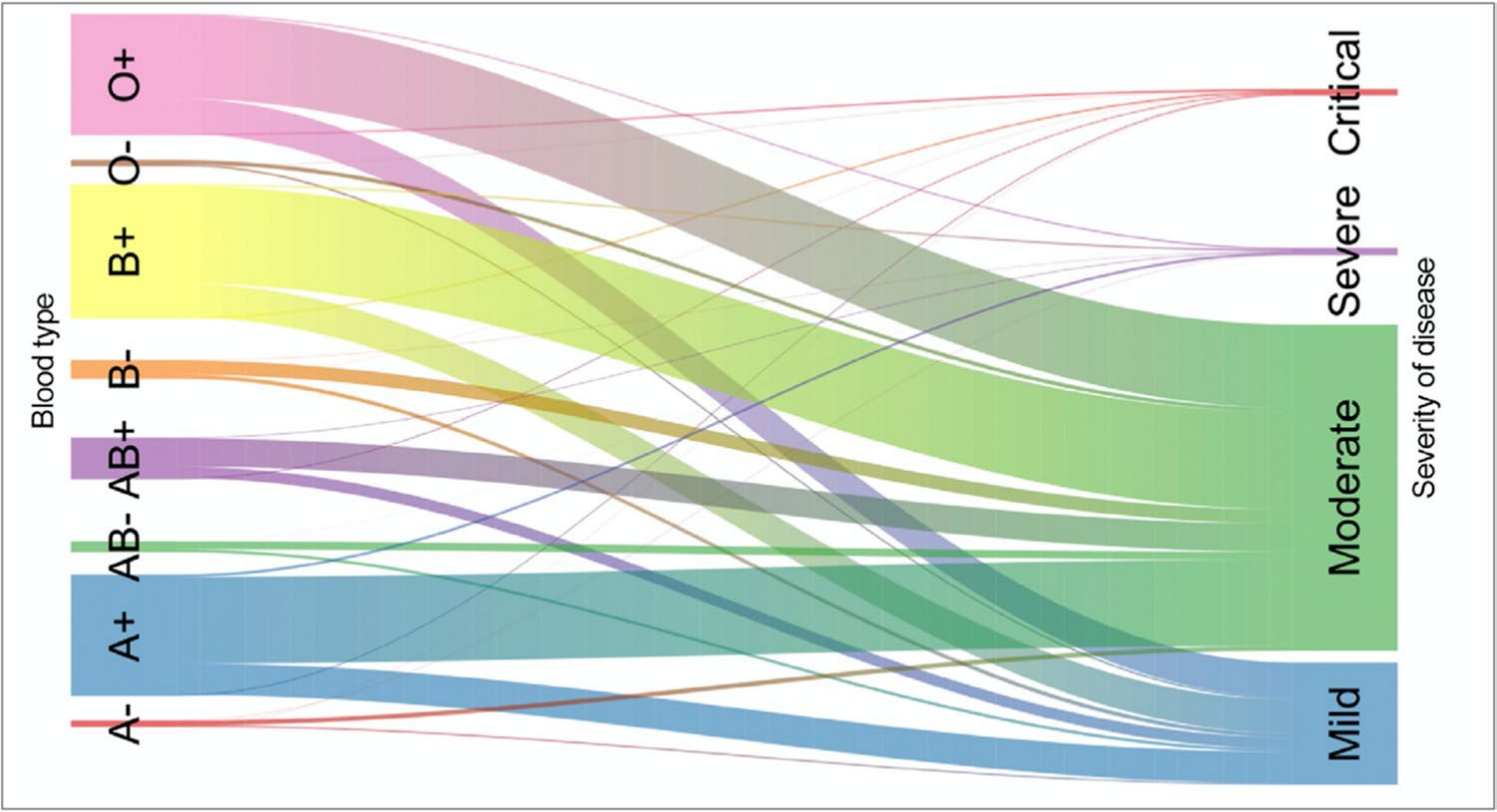
Flow diagram (Sankey plot) from Majeed et al. (2021) illustrating the distribution of mild, moderate, and severe COVID-19 among the ABO blood groups as represented by different colors in a large study from Iraq. The width of the lines corresponds to the percentage of the different subgroups. Available under a Creative Commons Attribution License

Less than 1 month after this latter study, another preprint on possible associations between ABO phenotypes and the risk of either contracting SARS-CoV-2 or the severity of COVID-19 appeared. In their study on US Americans, Zietz and Tatonetti (2020a) found a higher proportion of blood group A and a lower proportion of blood group O among COV + patients compared to COV − , though in both cases the result was significant only in Rh positive blood types. However, decreased odds for contracting the virus for group O and increased odds for A were suggested by the results of this study. In contrast, no significant association of the ABO blood group with intubation or death was noted (Table 1). Zietz and Tatonetti have published several updates of their study (Zietz and Tatonetti 2020b; Zietz et al. 2020a,b). With a growing number of patients included, previously postulated differences between blood groups with regard to the risk of infection were getting smaller. As for the severity of the disease, even a small advantage for A was seen (Fig. 2).

Problems with a study design became apparent from a study by Ellinghaus which was first published as a preprint (Ellinghaus et al. 2020) and later appeared in the New England Journal of Medicine (Severe Covid-19 GWAS Group et al. 2020). To the best of our knowledge, this was the first genome-wide association study (GWAS) searching for factors of the host genome that may influence either infection by SARS-CoV-2 or the severity of COVID-19. The results of the study pinpointed two interesting regions of the human genome with one of them covering the ABO locus. Nevertheless, this GWAS was based on a comparison between COVID-19 patients who needed oxygen supply and the general population, thus not allowing to distinguish between the risk of contracting the virus and the outcome of such an infection (Fig. 1). Criticism regarding such an interpretation of the study has been raised, e.g., by Boudin and Dutasta (2021) and by Bullerdiek (2020b).

In the following months, a number of reports on an association of ABO blood groups with either the risk of infection or the severity of disease appeared, which are included and summarized in Table 1. Almost all large studies failed to show a significant association of ABO blood groups with disease severity which is suggested by Ellinghaus et al. (2020) and the Severe Covid-19 GWAS Group (2020), respectively (for example, see Fig. 2). On the other hand, many of these studies suggest a slightly decreased risk of infection for blood group O compared to non-O, though not all recent studies did confirm such an association (e.g., Boudin et al. 2020; Rahim et al. 2021). However, that association alone could also explain, e.g., the data obtained by Ellinghaus et al. (2020). Another interesting example is offered by the recent paper by Ray et al. (2021). Aimed at demonstrating a possible association between disease severity and ABO-phenotype, they carried out an analysis restricted to 7071 persons who tested positive for SARS-CoV-2, which did not reveal an association between blood group and the risk for severe illness or death. However, analyzing all 225,556 persons, including those with a negative SARS-CoV-2 test result, type O blood versus non-O was protective against SARS-CoV-2 positivity with or without severe illness or death. This is exactly what can be expected if an association with the risk of getting infected exists, but once being infected, the ABO phenotype does not influence the likelihood of more or less severe disease. Accordingly, data from a recent meta-analysis do not confirm a relationship between ABO blood group and COVID-19 mortality (Boudin et al. 2020).

As to most recently published large studies, Anderson et al. (2021) have performed a case–control study including more than 10,000 individuals who were newly infected with SARS-CoV-2. This study did not reveal associations of blood type with disease susceptibility or severity, including viral positivity, hospitalization, or ICU admission. Also, compared with type O blood, type A was not associated with increased viral positivity, hospitalization, or ICU admission and types B and AB were not associated with worse outcomes than type O. Whereas these data concerning disease severity were confirmed, as to viral positivity, somewhat different data regarding SARS-CoV-2 susceptibility were reported by another large study: The host genetics initiative has recently published the results of three genome-wide association meta-analyses comprising data of up to 49,562 COVID-19 patients from 46 studies across 19 countries (The COVID-19 Host Genetics Initiative 2021). While the ABO locus was found to be associated with overall susceptibility to SARS-CoV-2 infection, no such association was noted with the progression to more severe COVID-19.

Obviously, the results of all studies as outlined in Table 1 cannot be combined in a meta-analysis because data evaluated differ from one study to the other. On the other hand, it may be helpful just to get an impression of the number of patients involved in studies claiming an association between ABO blood groups and the severity of COVID-19 and, on the opposite, of those studies that failed to support such an association. Omitting studies on self-reported data and GWAS, studies that did not show an effect of A on disease severity included 33,815 patients with different severity of COVID-19 or even just an infection without clinical symptoms. In contrast, studies revealing an association of blood group A with worse prognosis included 3963 patients.

In summary, available data are complex and do not offer convincing evidence for an association between ABO blood groups and severity of COVID-19. Nevertheless, this is only poorly reflected by the most recent reviews. Le Pendu et al. (2021) indirectly supports an association because some clinical findings associated with blood group A are also known to be associated with the severity of COVID-19 (see also, e.g., Nauffal et al. 2021). Another recent review (Liu et al. 2021) also argues for an association between disease severity and ABO blood groups but does not consider all of the recently obtained data.

## ABO blood groups and the risk contracting SARS-CoV-2

**Fig. 3:**
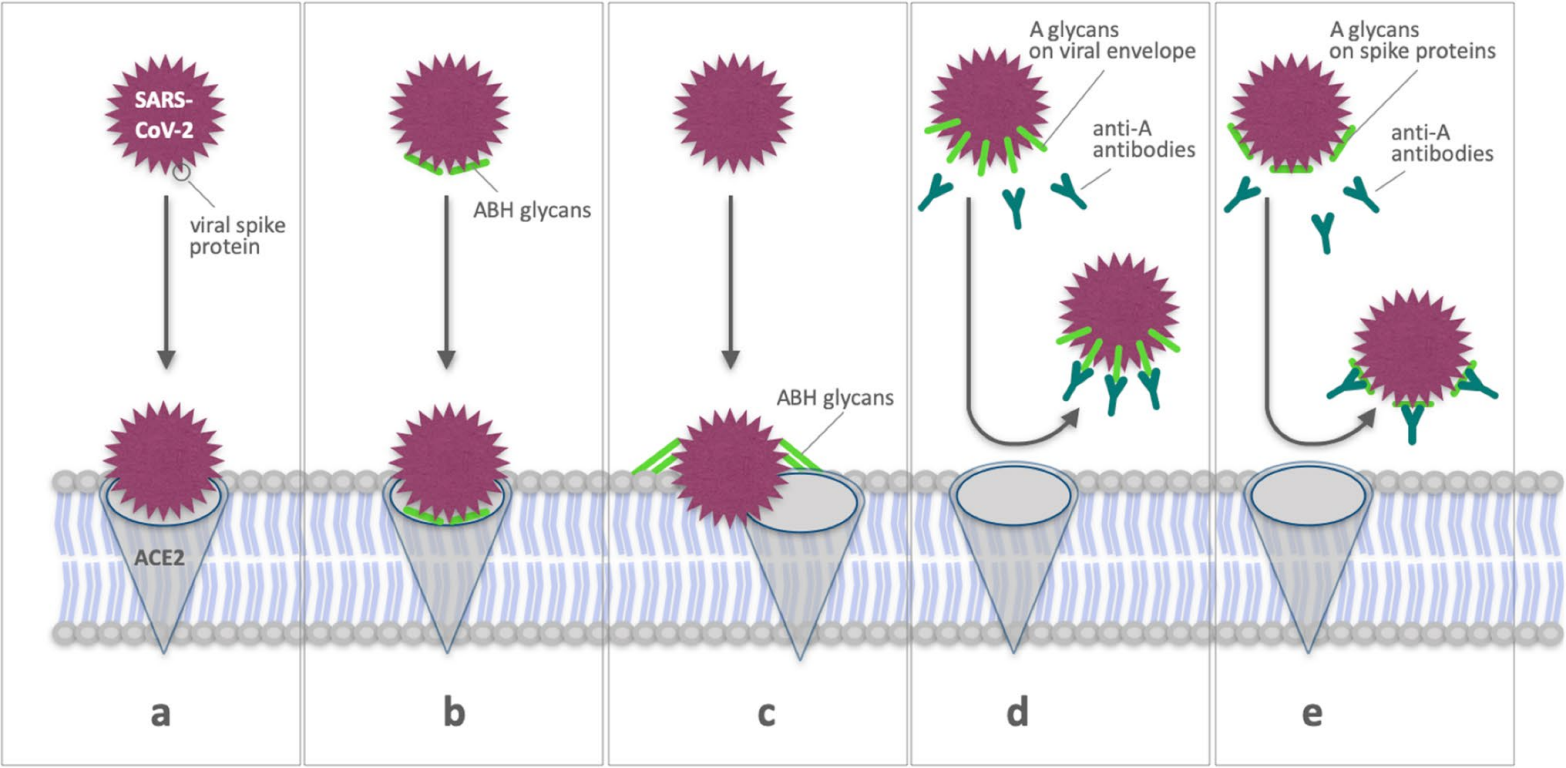
Schematic illustration of possible mechanisms that are discussed to explain a higher risk of blood type A individuals or a relative protection of O-type individuals to contract SARS-CoV-2. **a** Canonical interaction of viral spike protein with ACE2, **b** ABH glycans either associated with ACE2 or with the virus particle facilitating the interaction between viral spike protein and ACE2, **c** ABH glycans on the membrane of the target cell acting as non-canonical receptors, **d** anti-A antibodies blocking the interaction between ABH glycans attached to the viral surface and ACE2, and **e** anti-A antibodies binding to ABH glycans attached to the viral spike protein blocking virus-host cell contact thus reducing the risk of viral entry

In contrast to the severity of COVID-19, there is some evidence that the risk of contracting SARS-CoV-2 may depend on the ABO phenotype. Accordingly, a meta-analysis exclusively addressing the risk of infection presented by Golinelli et al. (2020) indicates that SARS-CoV-2 positive individuals are more likely to have blood group A (pooled *OR* 1.23, 95% *CI*: 1.09–1.40) and less likely to have blood group O (pooled *OR* = 0.77, 95% *CI*: 0.67–0.88). To explain this association, several modifications of the canonical interactions between the virus and the host cell membrane (Fig. 3a) are conceivable. Several possible interactions may favor an infection or severe COVID-19 in group A or non-O individuals, respectively, as, e.g., reviewed by Goel et al. (2021). In general, two types of interactions have been proposed where either ABO antigens facilitate viral attachment (Fig. 3b, c) or anti-A antibodies inhibit the binding to the cellular receptor (Zhang, Garner et al., 2021) (Fig. 3d, e).

The steps which might be influenced are viral attachment and in the following viral entry (Lan et al. 2020; Wrapp et al. 2020). Evidence for this assumption is, e.g., offered by Wu et al. (2021) who were able to show that the SARS-CoV-2 RBD exhibited high preference for the type of blood group A ABO(H) antigens (type I) expressed on respiratory epithelial cells. Nevertheless, they concluded that this does not definitively demonstrates a direct contribution of blood group A to SARS-CoV-2 infection making future studies expanding upon these initial findings necessary (Wu et al. 2021). In general, ABH glycans also have been considered as influencing factors. They may be either present on the SARS-CoV-2 envelope (Watanabe et al. 2020), modifying the affinity of SARS-CoV-2 for ACE2R (Fig. 3d, e) or being present on target cells (Fig. 3b, c), serving as lower affinity alternative or co-receptors for SARS-CoV-2 or binding other viral envelope structures. Of note, however, Schetelig et al. (2021) assume that the A epitope itself is not able to facilitate viral entry. Accordingly, they did not identify a consistent impact of ABO blood groups on the severity of COVID-19.

As an alternative explanation, anti-A and anti-B antibodies have been considered as protecting factors which by binding to their respective antigens expressed on the viral envelope or its spike protein may prevent infection of target cells. In general, while the spike protein of SARS-CoV-2 can facilitate cell entry through well-known interactions between its receptor binding domain and ACE2 (Yan et al. 2021), it has been hypothesized that the receptor binding domains may interact with other host molecules, including blood group antigens thus contributing to disease susceptibility (Wu et al. 2021). Binding of SARS-CoV-2 proteins by anti-A antibodies may prevent viral entry into the lung epithelium, e.g., by blocking interactions between ACE2R (angiotensin-converting enzyme 2 receptor) and the virus.

However, to the best of our knowledge so far, none of these hypotheses has been supported by direct experimental evidence.

## Do mutations matter?

Many of the known SARS-CoV-2 key mutations characterized by modified significant biological functions such as transmissibility, viral spreading, and escape of natural or vaccine-induced immunity affect the viral spike protein (Zhou and Wang 2021). Certainly, the appearance of some viral mutants can be expected to change the game if they influence the interaction between viral structures and the host cell (Hoffmann et al. 2021). For example, newly emerging variants of SARS-CoV-2 may influence the interaction between the RBD of spike glycoprotein of SARS-CoV-2 and its cognate cellular receptor ACE2. This, e.g., has been shown by in silico network analyses of mutations of the receptor-binding motif which aimed at the detection of hotspot points for drug design and the inhibition of the spike-ACE2 interaction (Jafary et al. 2021; Ortega et al. 2021). In turn, these interactions may be modified by the ABO blood groups additionally as discussed before.

## Conclusions

It was repeatedly suggested that the ABO blood groups are associated with either SARS-CoV-2 infection or with more or less severe COVID-19. Reasonable hypotheses linking different molecular mechanisms with these associations have been proposed as well but so far no direct evidence favoring one such mechanism has been presented. Also, summarizing the available information on SARS-CoV-2 infection, the clinical course of COVID-19, and ABO blood groups, there is little evidence that the severity of COVID-19 does depend on the ABO blood type of infected individuals. On the other hand, as to susceptibility for SARS-CoV-2 infection, individuals with blood group A may carry a higher risk compared to blood group O individuals. Nevertheless, conflicting data do exist in this case as well. However, should an association between ABO blood groups and infection be confirmed, it will remain to be discussed if and how after initial infection spreading of the virus in the infected tissue as well as systemically is also affected. Even more complicated, these findings may depend on the dominating viral subtype and its mutations, respectively, and it should be noted that most data are from early times of the pandemic.

In general, while in the future these findings may be of relevance for prevention and treatment of the disease, there seems to be no clinical relevance at this time at all. In particular, knowledge of a patient’s ABO phenotype should by no means directly influence therapeutical decisions.

## Supplementary Information

Below is the link to the electronic supplementary material.

Supplementary file1 Fig. 1 The first two pages of the article by Emil von Dungern and Ludwig Hirschfeld (1910) where for the first time the genetics of ABO blood groups have been described. (TIFF 8436 KB)

## Abbreviations

COVID-19: Coronavirus disease 2019
*FUT1*: Gene encoding galactoside alpha-(1,2)-fucosyltransferase 1
GWAS: Genome-wide association study
ICU: Intensive care unit
OR: Odds ratio
RBD: Receptor-binding domain
SARS-CoV-1: Severe acute respiratory syndrome coronavirus 1
SARS-CoV-2: Severe acute respiratory syndrome coronavirus 2
