# Supplementary figures and images for: ABO blood groups and the risk of SARS-CoV-2 infection
Source: Protoplasma. 2022 Apr 1;259(6):1381–95. doi: 10.1007/s00709-022-01754-1 (PMC8973646; doi:10.1007/s00709-022-01754-1)

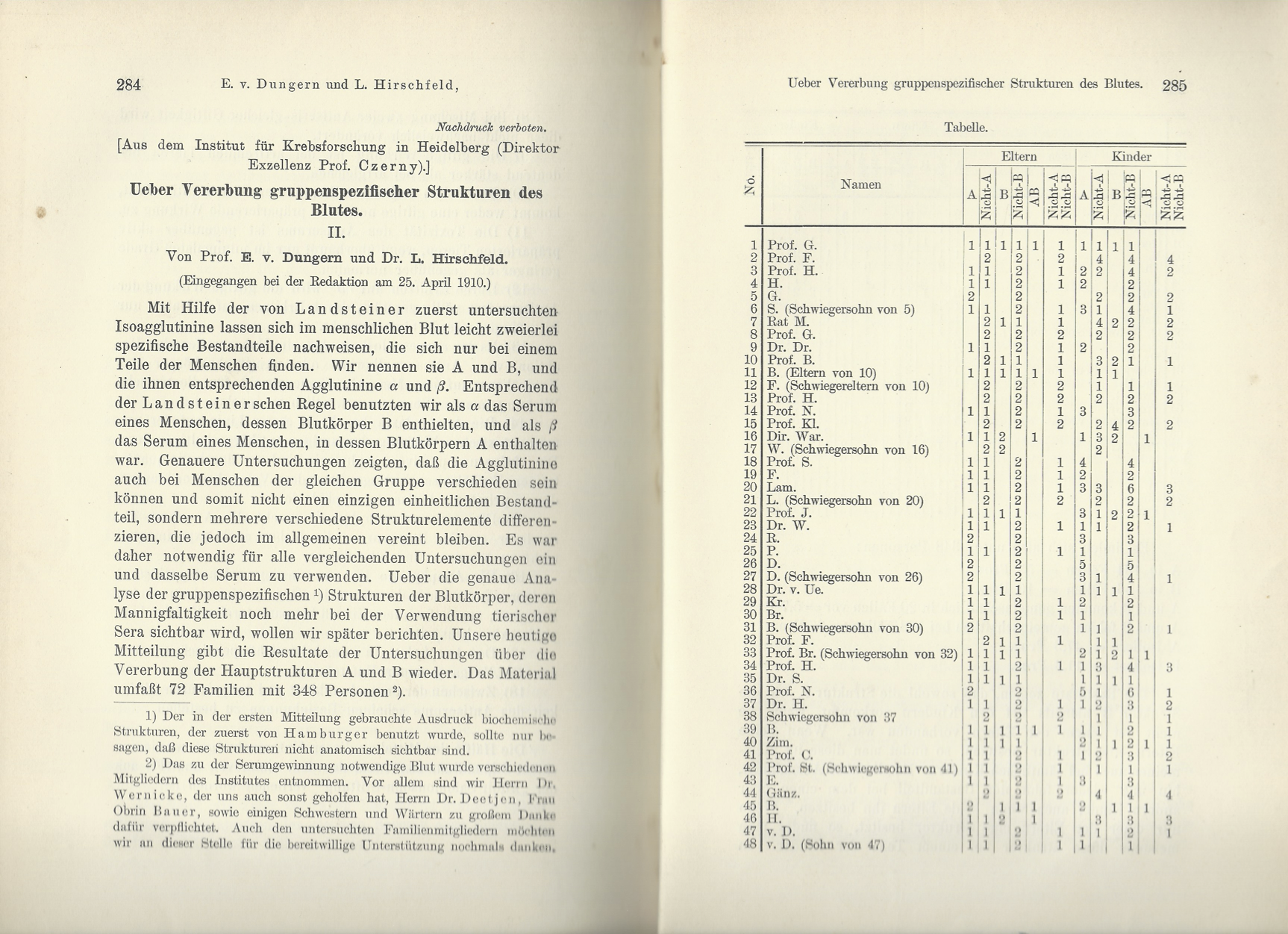

Supplement: Supplementary file 1 — Supplementary file1 Fig. 1 The first two pages of the article by Emil von Dungern and Ludwig Hirschfeld (1910) where for the first time the genetics of ABO blood groups have been described. (TIFF 8436 KB) [file 709_2022_1754_MOESM1_ESM.tiff]
